# Intensive Treatments in Adolescent Anorexia Nervosa

**DOI:** 10.3390/nu13041265

**Published:** 2021-04-13

**Authors:** Beate Herpertz-Dahlmann

**Affiliations:** Department of Child and Adolescent Psychiatry, Psychosomatics and Psychotherapy, University Hospital, RWTH, Neuenhofer Weg 21, D-52074 Aachen, Germany; bherpertz@ukaachen.de

**Keywords:** anorexia nervosa, adolescence, intensive care, inpatient treatment, day patient treatment, home treatment, hospitalization, refeeding, target weight

## Abstract

Approximately one-fifth to one-third of patients with adolescent anorexia nervosa (AN) need intensive care in the course of their illness. This article provides an update and discussion on different levels of intensive care (inpatient treatment (IP), day patient treatment (DP) and home treatment (HoT)) in different health care systems based on recently published literature. Important issues discussed in this article are new recommendations for the refeeding process and the definition of target weight as well as principles of medical stabilization and psychotherapeutic approaches. The pros and cons of longer or shorter hospitalization times are discussed, and the advantages of stepped care and day patient treatment are described. A new promising intensive treatment method involving the patient, their caregivers and the direct home environment is introduced. Parents and caregivers should be included in treatment research to foster collaborative work with the attending clinicians. There is an urgent need to evaluate the mid- to long-term outcomes of various intensive treatment programs to compare their effectiveness and costs across different health care systems. This could help policy makers and other stakeholders, such as public and private insurances, to enhance the quality of eating disorder care.

## 1. Introduction

Although anorexia nervosa (AN) may have onset in childhood, adolescence is recognized as a particularly important life stage in the development of this eating disorder (ED), with 20% to 40% of all new cases having their onset during this time [1]. There are conflicting results regarding whether the incidence and prevalence of adolescent AN have increased in recent years. While Scandinavian researchers found a significant rise in the incidence of AN in the first decade of this century, e.g., [2], this finding was not confirmed by others, e.g., [3]. (For a review, see [4].)

In adolescence, patients mostly fulfill classification criteria for the restrictive form of this ED. According to the data of the German national registry for AN, approximately 90% suffer from the restrictive type, but only approximately 10% from the binge/purge type [5]. Recently, the number of patients with atypical AN has risen sharply. According to the Diagnostic and Statistical Manual of Mental Disorders, Fifth Edition (DSM-5), all the criteria for AN are met in these patients, except that of a low body weight. In cases of atypical AN, the individual’s weight is within or above the normal weight range [6]. One of the main explanations is an increase in overweight and obesity in youth during recent decades resulting in the initiation of a strict diet to maximize weight loss. Recent studies comparing patients with AN and atypical AN have demonstrated a similar poor nutritional and medical status in both groups [7].

AN has the highest mortality of all mental disorders [8]. Some patients suffer from severe somatic or psychological comorbidity and require higher levels of care for their medical or psychological stabilization. This article summarizes current knowledge and clinical practice related to higher levels of care, e.g., inpatient treatment (IP), day patient treatment (DP) and home treatment (HoT). Across different health care systems, there is lack of agreement regarding how intensive treatment in AN should be practiced, which is a main point of discussion.

## 2. Inpatient Treatment

A rather dramatic increase in admission rates has been reported in several European countries; for example, hospital admissions nearly doubled for young people under 15 years old in Germany between 2000 and 2017 and have plateaued since then [9]; a similar trend was found in England [3,10]. The increasing admission rates may reflect an increasing incidence of AN in this age group (see above) or, more likely, a better knowledge and growing awareness by pediatricians and general practitioners of adolescent EDs [11]. Young people with AN have a higher probability of being admitted to the hospital than adults [3]. Moreover, the number of patients with atypical AN requiring medical hospitalization has risen sharply, accounting for approximately one-third of patients in hospital inpatient ED programs [7,12].

Recently, there has been some indication that the coronavirus pandemic has contributed to a strong increase in hospital admissions for adolescents with AN in Germany and other European countries [13], but also worldwide [14]. The patients’ explanation for the influence of the pandemic on AN incidence rates was that they had lost their daily structure of regular school visits and leisure activities (including sports) and instead developed a growing interest in food, diets and fitness exercises.

Notably, there are large discrepancies in admission rates and lengths of hospital stays between Anglo-Saxon countries and Western/Southern European countries, e.g., Germany, France and Italy [4]. However, there is some international consensus about medical and psychosocial indications for admission to IP (Table 1).

If IP treatment is necessary, adolescent patients should be cared for by a multiprofessional team experienced in the treatment of EDs. Ideally, this team will include a child and adolescent psychiatrist and/or pediatrician, a psychotherapist, a dietician, a physio- and/or occupational therapist and qualified nurses who can communicate with both the patient and their caregivers.

## 3. Refeeding

One of the main reasons for admission to the hospital is severe underweight or marked weight loss in a rather short period of time, such as in atypical AN. Thus, refeeding programs are necessary to allow restoration of a healthy body weight (for a clinical guidance to refeeding in AN, see [18]) and somatic stabilization. To re-establish normal eating habits, an individualized meal plan consisting of three main meals and three snacks is recommended (for a review, see [19]). Patients should be offered a nutritionally balanced diet; the calculation of caloric needs is based on age, gender, premorbid weight, activity level and the amount of weight increase necessary to achieve target weight (for more details, see [20]). A few years ago, clinicians were concerned about the so-called refeeding syndrome caused by realimentation-induced hypophosphatemia, with the possible consequences of renal and cardiac failure and neurological complications. In a recent randomized controlled trial (RCT) including 120 adolescents and young adults between 12 and 24 years, a high-calorie diet, defined as 2000 kcal/d at the beginning of refeeding and an additional 200 kcal every day, was compared to a lower-calorie diet, starting with 1400 calories and an augmentation of 200 kcal every other day. The main outcome in this study was medical stability, defined as a higher heart rate and a higher blood pressure (45 beats/min and >90 mmHg, respectively), a higher body temperature, less postural increase of heartbeat and orthostatic decrease in blood pressure and a higher body weight (75% of the mean body mass index (BMI) for the patient’s sex and age). Medical stability was restored significantly earlier in the high-calorie diet group than in the low-calorie diet group, and hospital stay was shortened by 4 days in the high-calorie refeeding group. Adverse events did not occur more frequently in the experimental high-calorie diet group [7]. The one-year follow-up results of this study have yet to be reported.

According to this and earlier studies, e.g., [21], faster realimentation than practiced previously should be recommended. However, according to our own experience, young girls (we refer to female patients, because most patients with AN are of female sex; however, male patients with AN are also included) often do not tolerate larger meal portions during the first days of the hospital stay. In comparison the patients in the study by Garber et al. [7], our patients are normally younger (15.06 vs. 16.4 years on average in our previous study [22]), which might prevent them from consuming larger amounts of food. In Garber et al.’s US study, refused food was replaced with a high-energy liquid formula, while we prefer our patients to start eating normal food. In our department, we normally take slightly more time than mentioned above to build up an age-adequate number of daily calories. We usually start at 1200 to 1400 kcal/day and increase the number of calories every second day by 200 kcal until the necessary intake is achieved. In the Garber study, the patients were only admitted for weight restoration, while in many departments in Europe, the aim of a longer hospital treatment is also to provide psychological support for patients to manage their ED behavior and cognitions. Thus, a therapeutic relationship is necessary, which might be impeded by the refeeding practices mentioned above.

Individual and group nutritional counseling by a dietician or an informed nurse for adolescents and their caregivers is recommended for adolescents with EDs to help them to meet their age- and development-appropriate dietary needs [17]. In our own experience, nutritional advice has proven to be very supportive for adolescents in re-establishing healthy eating behavior and gaining and maintaining weight.

## 4. Target Weight

There is still no consensus on the definition of a healthy body weight (and consequently a target weight) for age-appropriate growth and the resumption of menses. Two previous studies indicate the 25th age-adapted BMI percentile or 95% expected body weight (EBW) as the threshold for the reoccurrence of menses [23,24]. The EBW corresponds to the median age-adjusted BMI (50th BMI percentile) (%EBW is the observed BMI/EBW × 100). This definition is used mostly in the US.

An alternative method of determining target weight is using the adolescent’s BMI percentile history throughout her development. This method has the advantage of relying on multiple measurements and may account for AN-associated stunted height and individual differences in BMI. In a rather large study including 103 ED treatment providers, mostly from the US, approximately 40% used growth curve data and thus an individualized approach [25]. Against the background of an increasing prevalence of atypical AN [26], with weight loss mostly starting from a higher original weight, the determination of an individual target BMI may be more effective for growth and the resumption of menses than a standardized target weight. A higher BMI at discharge from IP seems to increase the likelihood of health maintenance [27,28,29].

## 5. Medical Stabilization

Starvation may be accompanied by severe physical changes, e.g., of the cardiovascular, gastrointestinal, metabolic and blood systems (for a review, see [30]). Although refeeding and weight gain will relieve most somatic problems, additional medical support, such as electrolyte substitution (in patients with excessive vomiting or laxative abuse), compensation of dehydration and restoration of a normal body temperature, must be considered. Many patients suffer from impaired gastric emptying and/or severe obstipation, which sometimes must be medically treated during early refeeding with prokinetic or osmotic agents, respectively. In contrast to many hospital programs (for a review, see [31]), we do not support bed rest for patients or transport in a wheelchair (apart from debilitating serious medical states). In a small RCT investigating the effect of regular exercise (twice daily jumping activity), the time period to vital sign stabilization was shorter in the exercising patients than in the nonactive control group and did not slow weight gain [32]. In another open trial with 29 patients with AN, BMI significantly increased, while ED and exercise dependence scores significantly decreased [33].

## 6. Psychotherapeutic Approaches

In addition to medical and nutritional stabilization, psychotherapy is one of the main “pillars” of IP.

However, providing individual and/or group psychotherapy is not a characteristic feature of only IP, as it is equally practiced in outpatient treatment, DP and HoT. First-line psychotherapeutic strategies in adolescent AN are family-based treatment (FBT) [34], adolescent-focused treatment [35] and cognitive–behavioral therapy for adolescents (CBT-E), e.g., [36].

FBT is the most studied treatment in adolescent AN and has the largest body of evidence regarding the three interventions mentioned above. Although it is mostly practiced in outpatient care, there have been some recent studies on the outcomes of FBT in inpatients with AN. A retrospective Canadian cohort study including 153 female adolescents admitted over a 5-year period with an average duration of stay of approximately 7 weeks examined the outcome at discharge defined by weight change and several psychological parameters. There was significant weight gain and an improvement in psychological well-being, although some patients displayed ongoing suicidality at discharge [37]. In a small Norwegian study with an average follow-up duration of 4.5 years, nearly two-thirds of the former inpatients who had received FBT during a hospital stay of approximately 21 weeks achieved a normal body weight [38].

Interestingly, many adolescents desire to have additional separate sessions without their caregivers [39].

Similarly, CBT was applied to a sample of 150 patients, including 74 adolescents who were provided with IP followed by DP (13 and 7 weeks, respectively) and who were reassessed after 60 weeks. The patients’ EDs and general psychopathology significantly improved, while their BMI was still in the lower normal range [40].

However, according to the conclusion of a recent survey in IP for adolescent AN, the role and effect of psychological interventions in addition to those of weight gain could not be evaluated [41].

## 7. Benefit of Hospitalization and Length of Stay in Adolescent AN

In a very recent evaluation, the impact of weight gain during intermittent hospitalization for medical stabilization in three RCTs comparing different psychotherapeutic interventions was investigated, indicating that weight gain during hospitalization added little to weight restoration at the end of treatment. However, the time of hospitalization was short and varied between 6 and 23 days [42]. Madden et al. [43] explored the effectiveness of longer hospitalization (37 days) compared to that of a shorter stay for medical stabilization (22 days). All patients participated in a nasogastric refeeding program. Both treatments were followed by 20 sessions of FBT. At the 12-month follow-up, there was no significant difference in hospital bed use after discharge between the two groups, but health care costs were significantly lower in the “only medically stabilized” group. However, the length of hospital stay between both treatment methods differed by only 15 days; other differences in treatment approaches were not described. Unfortunately, the effect of nasogastric feeding in comparison to that of “autonomous eating” on longer-term treatment outcomes has never been systematically investigated. In a survey on inpatients’ and their caregivers’ perspectives on treatment, patients—in contrast to their caregivers—desired more gradual weight gain and longer admission periods [39].

It is certainly not advisable to compare the short-term effect of intermittent hospitalizations aimed at medical stabilization mostly practiced in the US and the UK to that of psychiatric or behavioral hospitalization for adolescent AN in several central European countries. Long-term international studies are needed to evaluate the benefit for the individual, their caregivers and society, including health care costs. 

## 8. Day Patient Treatment

Recent studies have demonstrated that DP or partial hospitalization is often a valuable alternative to IP in medically stable adolescents. However, studies on its effectiveness are still scarce, and DP programs vary considerably in daily hours and length of stay. Treatment offers range from 4 to 7 days per week; duration of treatment is between 3 and 18 weeks (for a review, see also [4]).

A previous systematic review by Friedman et al. [44] evaluated partial hospitalization programs in adolescents with AN across six open studies and concluded that DP was effective in improving weight gain and eating behavior. This finding was confirmed by a Spanish study of an intensive DP program of 11 h per day [45].

In another rather large clinical trial including 326 participants, the majority of whom were adolescents, the implementation of FBT in a partial hospital program resulted in a significantly lower rehospitalization rate in comparison to that of a previous program without FBT [46]. Simic et al. [47] retrospectively evaluated the hospital charts of 105 patients with adolescent restrictive AN who had been admitted to a DP clinic after FBT-oriented outpatient treatment had failed. The average duration of DP was approximately one month, followed by outpatient treatment in the same service; outcome assessment was conducted six months later and again at discharge from the Child and Adolescent Eating Disorder Service. Several clinical parameters, such as the difference in BMI between admission and follow-up, eating behavior, comorbid psychiatric symptoms, motivation to change and quality of life, improved significantly.

In our own stepped care model, 172 patients were either randomized to DP after 3 weeks of IP for medical stabilization or to continued IP. Patients in the DP arm received the same treatment components as those in the IP arm. At the 1-year follow-up, DP was not inferior to IP with respect to weight gain. Moreover, DP proved to be safe and less costly than IP. Patients in DP showed better psychosexual development and mental states than those in IP [48] At the 2.5-year follow-up, patients treated in DP had a higher weight and fewer rehospitalizations than those in the IP group [49]

Since the publication of this RCT, the implementation of DP treatment in routine care has made good progress, and several trials have been initiated to explore its effects on outcomes in adolescent and adult AN (e.g., DAISIES by King’s College London) [50].

## 9. Home Treatment

The data on HoT in mental illness, although increasing, are limited. The available studies on this kind of community-based treatment in psychiatric disorders indicate its general safety and feasibility, even for severely ill patients [51]. In a previous systematic review of research with mentally ill adults, adolescents and children, HoT was found to be effective in decreasing the number of hospitalizations and improving cost-effectiveness [52]. Another review including six RCTs comparing IP with specialist outpatient treatment, multisystemic therapy, DP, intensive home treatment and supported discharge service demonstrated similar improvements of illness symptomatology in the latter services as in IP. The authors reported lower costs, greater patient satisfaction and fewer rehospitalizations for the treatment models other than IP [53].

However, to our knowledge, with the exception of the study by Boege et al. [51], which included five patients with AN, no previous studies have explored the effectiveness of HoT in adolescents with AN.

In our pilot study, we investigated the course and outcome of AN during a stepped care treatment program of IP followed by HoT until a 1-year follow-up. We included only patients who lived with at least one parent, within a 1 h commute from our department, who did not have severe physical or mental comorbidity and who had an IQ ≥ 80. Moreover, only patients admitted to the hospital for the first or second time because of AN were included. Due to the experimental character of this treatment, we did not include patients with chronic forms of AN.

The HoT procedure can be described as follows. After 5–8 weeks of IP, patients are discharged home. During the IP period, they and their caregivers are prepared for the new treatment mode. With the help of a psychoeducational group program, parents are informed about the consequences of starvation and their role in the treatment of AN. In addition, on the ward, parents must take part in joint family meals and psychotherapeutic sessions with their daughters. As soon as the patients are medically stabilized, they spend the weekend at home and are supervised and supported by their caregivers to comply with their meal plans. At the time of discharge from the ward, they must be able to eat autonomously, and the target weight is determined. The exclusion criteria for HoT after the end of IP are extreme exercising, frequent vomiting or laxative abuse, insufficient weight gain during IP and ongoing severe physical and mental comorbidity (see above). HoT is conducted following a tiered approach by an experienced multiprofessional team. The team comprises the individual therapist (child and adolescent psychiatrist or psychotherapist) already known to the patient and their parents from IP treatment, a nutritional therapist, an experienced nurse and an occupational therapist who is experienced in body image therapy. The whole team is supervised weekly by an experienced child and adolescent psychiatrist. During the first and second months after discharge, the patient and their family are visited three to four times a week; in months 3 and 4, the number of visits decreases to two and one visit per week, respectively. The professionals of each discipline have a role in this new treatment method; however, the frequency of visits by each professional depends on the individual patient’s and caregivers’ needs. Some families need more advice with nutritional problems, while others need more support regarding family conflicts. In any case, the patient is regularly seen by their individual therapist. Before the beginning of HoT, parents must agree to participate in at least one weekly visit of the team. During the course of HoT, the focus changes from management of food intake and weight gain to social reintegration and age-adapted autonomy (for a review, see also [22]), which is an important objective in the treatment of adolescent AN.

The evaluation in our pilot study demonstrated good clinical effectiveness of HoT for patients and caregivers. Most of our patients achieved their target weight and maintained this weight at the 1-year follow-up. ED psychopathology declined significantly and did not differ from the improvements achieved in our DP trial [22,48]. Interestingly, motivation to change increased significantly during HoT and remained stable thereafter until the one-year assessment point [54]. Moreover, the skills of the caregivers to handle their daughters’ EDs, measured by different instruments, such as the Accommodation and Enabling Scale for Eating Disorders [55], significantly increased, and their burden and depressive symptoms decreased.

Our explanations for the effectiveness of HoT are as follows. First, the formation of habits plays an important role in the development of a chronic ED [56]. While at the beginning of an ED, behavior patterns such as fasting or exercising are followed by an (internal) reward such as the desired weight loss and/or a feeling of self-control and self-satisfaction, this self-affirmation subsides during the course of the illness [57]. Instead, ED behavior becomes self-perpetuating and self-reinforcing. The longer the duration of illness, the more pronounced ED habits are [58]. Most ED habits manifest at home; thus, rigid and ritualized eating behavior and exercising may be interrupted by HoT at an early stage of the illness. A second reason could be the strengthening of the caregivers’ role during HoT. In their “cognitive interpersonal maintenance model”, Schmidt and Treasure hypothesized that the reaction of caregivers toward the EDs of their children might unintentionally reinforce ED symptoms; e.g., the insecurity and frustration of the caregivers might foster overprotection and criticism [59]. As mentioned below, counseling and support by professionals [60,61] are among the most urgent needs expressed by parents. Practical assistance at home and a greater feeling of security might enable caregivers to interact with their child in a more constructive and supportive manner.

However, our study was only a pilot study, and the findings must be confirmed by an RCT with a much larger sample size. In addition, we understand that HoT will not be helpful for all patients with AN, e.g., for those with a chronic course of the disorder or with severe somatic and mental comorbidity, such as a full-blown obsessive-compulsive disorder. However, for those with a less habitualized AN, HoT might be a very helpful strategy to prevent chronicity.

## 10. Perspectives of Patients and Their Caregivers

A recent qualitative study conducted an exploration with 19 adolescents and young adults between 16 and 25 years, the majority of whom suffered from AN, to gain insight into the patients’ and caregivers’ needs and desires for the organization of treatment in EDs. One of the key themes was to move away from a mainly weight-focused to a more holistic and individualized treatment; another focus was family and peer support [61,62]. In an earlier study, the most frequent request of caregivers was “counseling and support” by a professional [60].

A considerable percentage of young people view support from their caregivers and siblings to be important for overcoming their illness. Other patients underscore the necessity to keep in touch with friends during treatment and reconnect if friendship is hampered, while others emphasize the necessity to develop new friendships [63].

## 11. Conclusions

Approximately 20% to 30% of all patients with AN need intensive treatment (IP, DP or HoT) to improve or recover. There are significant differences among health care systems and different indications for hospital treatment, resulting in the varying availability of treatment options without sufficient evidence of which treatment setting works for whom. According to the viewpoint of Kaye and Bulik (2021), there is not only a crisis of care for AN in the US, but also in several European countries [64]). In addition, patient and caregiver perspectives are often neglected. Both perspectives should be included in research planning to determine which gaps in intensive treatment deserve priority attention. Ultimately, (transcontinental) studies evaluating mid- to long-term outcomes of different ED programs implemented by different health care systems are urgently needed to compare the effectiveness and costs of the programs (including personal and societal tolls of a chronic illness). An evidence-based agreement on the most important components of intensive care in adolescent AN would help policymakers and public insurance programs to foster treatment programs which are most helpful to improve the mid- and long-term outcome of AN. Otherwise, we will rely only on our own experiences and beliefs and continue “to swim in our own little national soup” without knowing which treatment strategy best meets the requirements of our patients. 

## Figures and Tables

**Table 1:** Indications for admission to hospital for adolescents with anorexia nervosa (AN) [15,16,17].

Body mass index below the 3rd age-adapted percentile or marked weight loss (e.g., 20% of original body weight in six months) or complete food denial during the previous days
Cardiac risk factors (hypotension (<80/50 mm Hg) or bradycardia (<50 beats/min), ECG changes (e.g., prolonged QRS interval, arrhythmia)
Low body temperature (<35 °C)
Dehydration and refusal to drink
Metabolic risk factors such as hypoglycemia, electrolyte disturbances, liver or kidney affection
Frequent vomiting or severe laxative abuse
Severe comorbid mental disorder, suicidality
Insufficient weight gain despite adequate outpatient treatment
Severe conflicts with caregivers

## Data Availability

Not applicable
